# The WHO classification of haematolymphoid tumours: response to Swerdlow et al.

**DOI:** 10.1038/s41375-022-01694-y

**Published:** 2022-09-28

**Authors:** Ian A. Cree

**Affiliations:** International Agency for Research on Cancer (IARC), World Health Organization, 150 Cours Albert Thomas, 69372, Lyon, Cedex 08 France

**Keywords:** Haematological diseases, Diagnosis

## To the Editor:

I am grateful to the authors of this letter for their continued interest in this subject over many decades, and their past support for the WHO Classification of Tumours. Governance matters, and it was quite clear that the CAC mechanism did not meet our requirements and could not continue alongside the editorial board for the reasons given in my editorial [1]. While the correspondents may find this disappointing, the new edition of the Haematolymphoid volume has been thoroughly reorganised and rewritten with the help of 420 authors and editors with multidisciplinary expertise and worldwide representation, including many members of the European Association for Haematopathology and the Society for Hematopathology, according to our published methodology [2, 3]. The Haematolymphoid Tumours volume (5th ed.) is now published online in beta format [4] and readers can judge for themselves how we’ve done, with the opportunity for feedback from our website. Personally, I am very grateful to all involved who have worked tirelessly to achieve an excellent hierarchical classification as well as a level of detailed description of these disorders that have never been bettered.

## Disclaimer

The content of this article represents the personal views of the author and does not represent the views of the author’s employers and associated institutions. Where the author is identified as personnel of the International Agency for Research on Cancer/World Health Organization, the author alone is responsible for the views expressed in this article, and does not necessarily represent the decisions, policy or views of the International Agency for Research on Cancer/World Health Organization.
